# Anthropometric and Biochemical Markers as Possible Indicators of Left Ventricular Abnormal Geometric Pattern and Function Impairment in Obese Normotensive Children

**DOI:** 10.3390/diagnostics10070468

**Published:** 2020-07-10

**Authors:** Filippina Giannisi, Anastasia Keivanidou, Ioanna Sakellari, Sofia Balala, Maria Hassapidou, Areti Hitoglou-Makedou, Andreas Giannopoulos

**Affiliations:** 1 2nd Pediatric Department, Aristotle University of Thessaloniki, AHEPA General Hospital, 54636 Thessaloniki, Greece; keivanidou@yahoo.com (A.K.); aretimak@med.auth.gr (A.H.-M.); agianop@auth.gr (A.G.); 2 Department of Nutritional Sciences and Dietetics, School of Health Sciences, International Hellenic University, 57400 Thessaloniki, Greece; ioanna_sakel@hotmail.com (I.S.); balalasofia@yahoo.com (S.B.); mnhass@gmail.com (M.H.)

**Keywords:** childhood obesity, cortisol, diastolic function, hs-CRP, tissue Doppler, waist-to-height ratio

## Abstract

Εmerging data indicate that various effects of obesity on the cardiovascular system can be evident during childhood. The aim of this study was to detect early changes in left ventricular structure and function in obese normotensive children and explore possible associations of these changes with anthropometric and biochemical parameters. Normotensive 8–11-year-old obese and normal weight children were included in the study. They all underwent anthropometric measurements, laboratory tests, and echocardiography study by conventional and tissue Doppler to assess geometric pattern and function of left ventricle. Statistically significant differences in most anthropometric and metabolic parameters were noticed between groups. Obese children showed higher left ventricular mass index (LVMI) (40.05 ± 9.44 vs. 28.31 ± 6.22), lower E/A ratio (1.76 ± 0.33 vs. 2.08 ± 0.56), and higher E/e’ (6.04 ± 1.13 vs. 5.43 ± 0.96) compared to lean peers. Waist-to-height ratio and hs-CRP correlated significantly with E/A in the obese group. Left ventricular hypertrophy was present in 47.2% of obese children and eccentric was the prominent type. Waist-to-height ratio and serum cortisol levels in plasma increased the odds of having any type of abnormal ventricular geometric pattern. Echocardiographic evaluation of left ventricle and diastolic function could be considered for obese normotensive children based on waist-to-height ratio, hs-CRP, and serum cortisol.

## 1. Introduction

Childhood obesity, defined as abnormal or excessive fat accumulation that presents a risk to health, is one of the most challenging health problems around the world, with increasing rates in developed but also in developing countries during the last decades [1]. It is estimated that 40 million children under the age of 5 years and more than 330 million children and adolescents aged 5–19 years were overweight or obese in 2016 [2]. It seems that a high percentage of obese children tend to remain obese through adolescence and adulthood [3] with higher cardiovascular risk [4]. Overweight or obesity in adolescence may account for as much as 20% of cardiovascular deaths and 25% of deaths from coronary heart disease in midlife [5]. Incidence of comorbidities such as hypertension, insulin resistance, type 2 diabetes, non-alcoholic liver disease, and dyslipidemia seems to be higher in obese teens compared to normal weight peers [6].

Apart from clustering of metabolic risk factors, obesity can cause changes in left ventricular (LV) size and diastolic function that can be already detected at an early age. Left ventricular hypertrophy (LVH), defined as an increase in left ventricular mass (LVM), is an independent risk factor for cardiovascular morbidity and mortality [7,8] and seems to be prevalent in obese children and adolescents [9,10,11]. The association between obesity and left ventricular mass (LVMI) has been evident since 1991, when results from the Framingham Heart Study showed that BMI remained a strong predictor of LVM and LVH in adults, after adjusting for age and BP [12]. Body composition, stage of sexual maturation and gender affect LVM in different ways, while not only excess of fat but also central distribution of it plays a significant role [13]. As regards to diastolic function in obese children, studies are inconclusive, with some of them indicating decreased LV early relaxation compared to matched lean controls [14,15,16] whereas other researchers did not confirm any differences [17,18]. Discrepancies in the results could be attributed to different ultrasound techniques used, sample variability, and definitions for overweight and obesity.

The mechanisms implicated in the pathophysiology of altered LV structure and function in obesity have not been completely clarified. Increased metabolic activity caused by excess of adipose tissue leads obese individuals to volume overload and a subsequent alteration of LVM and geometric pattern of LV in response to hemodynamic changes [19]. Elevated blood pressure, present in up to 23% of obese youth [20], is also a well-studied risk factor for LVH due to increased afterload. Although a link between LVH and early relaxation abnormalities seems reasonable, an association between LVMI and diastolic parameters has not been demonstrated in most studies [21,22]. Hence, other non-hemodynamic mechanisms may be responsible for impaired diastolic indices in obese children compared to lean peers. Progressive obesity leads adipocytes to loss of storage ability, eventually resulting in increased levels of circulating free fatty acids, lipid deposition on ectopic tissue (pancreas, liver, heart), and lipotoxicity [23]. Abundant visceral fat in obese subjects is also dysfunctional leading to increased synthesis of pro-inflammatory cytokines, such as leptin and resistin, and decreased secretion of protective insulin-sensitizing adiponectins [19]. This “adiposopathy” results in insulin resistance and hyperinsulinemia, glucotoxicity, increased angiotensin synthesis, and activation of the sympathetic nervous system, contributing further to myocardial dysfunction [24].

Aim of our study was to assess LV structure and diastolic function in obese prepubertal normotensive children by means of conventional and tissue Doppler imaging (TDI) and to investigate the possible association with anthropometric parameters, metabolic factors and chronic inflammation measured by high sensitivity C-reactive protein (hs-CRP).

## 2. Materials and Methods

Our sample was prospectively selected from children visiting the outpatient clinic of our Pediatric Cardiology Department for routine checkup and issue of health certificate, from September 2014 to October 2017. Inclusion criteria were age 7.5–11.5 years old, puberty stage Tanner 1 and BMI above 95th percentile according to IOTF cut off levels [25] for the case group or below 85th percentile for the control group. Exclusion criteria were cardiac disorders, hormonal disorders or any other chronic condition. Children and their guardians were fully informed about the protocol, approved by the Bioethics Committee of the Medical School of Aristotle University of Thessaloniki and written informed consent was given by parents (3850, 14-06-2012).

All subjects underwent full physical examination and complete medical history was recorded. Anthropometric measurements were taken twice from the right side of the body, with children standing still in erect position, by the same trained examiner. Height (in meters) was measured to the nearest 0.1 cm with a standard stadiometer (Seca 213, 22,089 Hamburg, Germany), weight (in kg) to the nearest 0.1 kg with a standard scale (Seca 813, 22,089 Hamburg, Germany) and various body circumferences to the nearest 0.1 cm with a standard measuring tape (Seca 201, 22,089 Hamburg, Germany), after removal of shoes and heavy clothes. Neck, waist, and hip circumferences were obtained by placing the tape just below the thyroid cartilage, on the midway between the lowest ribs and the iliac crest and at the level of great trochanters, respectively [26,27]. BMI was assessed from weight and height using Quetelet’s equation (weight [kg]/height[m^2^]). Additionally, skinfold thickness at three sites were measured with a standard caliper (Slimguide Skinfold): Triceps fold (back of the mid-upper arm), subscapular fold (below the shoulder blade) and suprailiac fold (on the middle of the distance between the lower rib and the top of iliac crest). Final value was the average of 3 consecutive cycles of measurements. Puberty status was assessed according to Tanner staging method by the same trained physician [28]. Waist-to-height ratio (WHtR) and waist-to-hip ratio (WHR) were also calculated [29,30].

Fasting blood samples were drawn between 8–9 am and the following biochemical markers were assessed: Glucose, total cholesterol (TC), high-density lipoprotein (HDL), low-density lipoproteins (LDL), triglycerides (TG), aspartate amino-transferase (AST), alanine aminotransferase (ALT), gamma-glutamyl transpeptidase (GGT), and uric acid (UA) by Cobas Integra 400 analyzer. High sensitivity C reactive protein (hs-CRP) by CRP latex agglutination test, insulin, cortisol, and thyroid-stimulating hormone (TSH) were measured by electrochemiluminescence immunoassay analyzer (ECLIA) and HbA1c (%) by high-performance liquid chromatography (HPLC-IFCC approved). Children with abnormal TSH were referred to an endocrinologist and were excluded from the study. Insulin resistance was estimated by calculating homeostatic model assessment of insulin resistance (HOMA-IR) according to the formula: Fasting insulin (μU/L) × fasting glucose (mmol/L)/22.5. Children with values ≥3.16 were considered insulin-resistant. [31].

Cardiac examination was conducted by the same experienced pediatric cardiologist. Firstly, blood pressure was measured after sitting quiet for 5 min, twice in both arms and in a leg, with an appropriately sized cuff, using a Dinamap monitor. BP values >90th percentile for age, height and gender [32] were considered elevated and patients were excluded, according to the study protocol. Echocardiography was performed afterwards by using a General Electric Vivid 3 medical system, with an appropriate for patient size probe, with the subject lying in the left lateral decubitus position.

M-mode parameters captured in the longitudinal axis included: Left atrial diameter (LAD), intraventricular septal thickness at diastole (IVSd), left ventricular end diastolic dimension (LVIDd), posterior wall dimension at diastole (LVPWd), left ventricular mass (LVM), intraventricular septal thickness at systole (IVSs), left ventricular end systolic dimension (LVIDs), posterior wall dimension at systole (LVPWs), and ejection fraction (EF). LVM was indexed (LVMI) to height in meters raised to the 2.7 power, as it is considered more reliable for pediatric patients [33]. Left ventricular hypertrophy (LVH) was defined by values above 40 gr/m ^2.7^ and 45 gr/m ^2.7^, for girls and boys respectively [33]. Relative wall thickness (RWT) was calculated according to formula: (IVSd+LVPWd)/LVIDd and values above 0.41 were considered abnormal, as it represents the 95th percentile [34]. LVH was categorized into four patterns, based on LVMI and RWT. Normal when both LVMI and RWT were within normal limits, eccentric hypertrophy when only LVMI was elevated, concentric hypertrophy when both LVMI and RWT were abnormal and concentric remodeling when RWT was high in the presence of normal LVMI [35]. Pulsed wave Doppler measurements of the mitral inflow obtained in the apical four-chamber view with the sample volume placed between the mitral valve leaflet tips included: early diastolic mitral velocity (E), late diastolic mitral velocity (A) and E/A ratio (indicator of LV diastolic function).

Tissue Doppler imaging (TDI) was also performed to assess diastolic function. The sample volume was placed at the medial (septal) and lateral (wall) mitral annulus and e’ and a’ velocities were measured. The average of velocities from both sites was taken into account. The ratio of early mitral flow velocity (E) to early diastolic velocity of the mitral annulus (e’) was used as a surrogate of LV filling pressure in early diastole, with high E/e’ ratio reflecting impaired relaxation. Measurements were obtained with minimal angle of incidence (angle between the direction of wall motion and the Doppler beam).

Continuous variables were presented as mean ± standard deviation (SD) and categorical variables as frequencies and percentages. Normality was tested using the Kolmogorov–Smirnov test. Differences in continuous variables between the study groups were assessed using independent sample *t*-tests and Mann–Whitney *U*-tests for normally and abnormally distributed data, respectively. Spearman correlation co-efficient (rho) was used to describe the correlation between continuous variables. Variables found to be correlated by any of the above tests were then entered in a multivariate regression model. Stepwise multiple regression analysis was used to determine which independent predictor variables explained a significant fraction of the variance of the dependent variables. Linear or logistic regression was used accordingly to the nature of the dependent variable. Not normally distributed variables were log transformed in order to be included in regression analysis. Statistical significance was set at *p* < 0.05. Statistical analyses were performed using the Statistical Package for Social Sciences 25.0 program.

## 3. Results

A total of 62 children were enrolled in the study and the anthropometric and biochemical characteristics of children are shown in Table 1 and Table 2. There were no differences in age and sex distribution between the 2 groups. However, statistically significant differences were revealed in all anthropometric parameters and biochemical markers except for total cholesterol, HbA1c and cortisol levels.

TSH was within normal limits across the sample. HOMA-IR was ≥3.16 in 38.9% of obese children. No differences in anthropometric, metabolic, and cardiac parameters between insulin resistant and the rest of obese children were noticed.

Systolic blood pressure was by protocol < 90th percentile, but was significantly higher in obese children. Diastolic blood pressure did not differ between groups.

Regarding echocardiographic findings (Table 3), structural parameters (LAD, IVSd, LVIDd, LVPWd, and LVMI) were significantly higher in obese children. Ejection fraction was within normal range and similar between 2 groups. Mitral inflow velocity, expressed by E/A ratio, was normal (above 1) in all children but significantly lower in the obese group (1.76 ± 0.33 vs. 2.08 ± 0.56).

TDI measurements revealed impaired diastolic relaxation in obese children expressed by a higher E/e’ ratio (6.04 ± 1.13 vs. 5.43 ± 0.96).

Correlations by Spearman coefficient (rho) for both groups of LVMI, E/A, and E/e’ with various parameters are shown in Table 4. When only obese children were analyzed then the only correlations maintained were those of LVMI with anthropometric parameters (BMI, WHtR, NC) and E/A with WHtR and hs-CRP levels. None of the recorded parameters correlated with E/e’ in the obese group. (Table 4).

**Figure 1:**
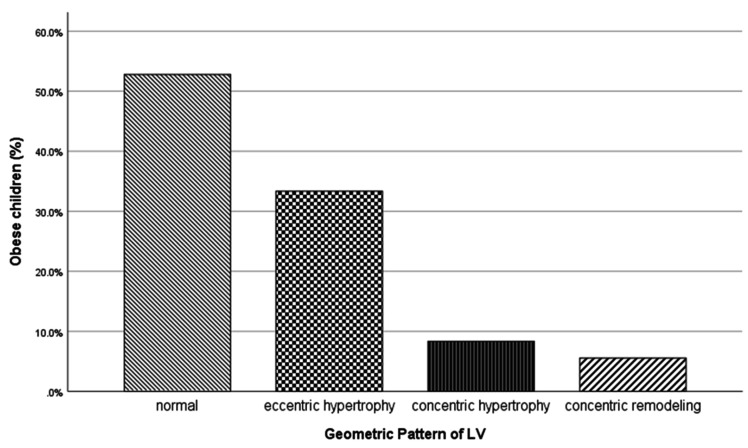
Left ventricular geometric patterns in obese children.

Among obese children 47.2% (12/36) had abnormal geometric pattern with a predomination of eccentric hypertrophy (Figure 1).

WHtR and the log transformed plasma cortisol concentration were statistically different between those with and without remodeling. By means of binary logistic regression in obese children, the estimated odds ratio for the presence of any type of abnormal geometry increased for every one unit increase of WHtR (OR: 1.296, 95% CI:1.046–1.605), *p*: 0.018) and log serum cortisol (OR:17.305, 95% CI:1.62–185.08, *p*: 0.018) (Table 5).

In a multiple linear regression model only hs-CRP was significant predictor of E/A (Beta = −0.727, *p* = 0.012) with adjusted R^2^ of 0.147.

## 4. Discussion

This study showed that changes in cardiac structure and diastolic function can be detected early in obese prepubertal children even in the absence of hypertension. Thickness of cardiac walls, left ventricular mass and indices of filling capacity of left ventricle, differed significantly between obese and lean subjects. Nearly 1 out of 2 children had some kind of abnormal pattern of LVH and the risk of remodeling increased in proportion to WHtR and serum cortisol levels. Diastolic function expressed by E/A correlated significantly with WHtR and hs-CRP levels.

Increased intraventricular septal thickness and higher LVMI in obese compared to normal weight children have been noticed previously in studies, varying in regard to sample size, blood pressure status, and puberty stage [16,36,37]. In our sample we tried to eliminate confounders like hypertension and puberty and still dimensions of left ventricle as well as LVMI were significantly associated to several anthropometric indices, confirming that obesity has an independent impact on cardiac structure. Interestingly, LVMI showed stronger relation to WHtR rather than BMI, probably implicating stronger influence of central adiposity [38,39], but this relationship was not verified in some studies [40,41].

Left ventricular hypertrophy has been traditionally considered a form of end organ damage in hypertensive population. Nearly half of the obese group met criteria for LVH, despite the fact that none of the children selected was hypertensive. This finding implies that excess of fat and consequent metabolic dysregulation may play a more important role in changes in left ventricle than hypertension in obese subjects [24]. Our data showed that elevated WHtR ratio and serum cortisol levels increased the odds for having any type of left ventricular remodeling. The possible role of cortisol excess has been investigated in patients with Cushing syndrome, in whom a high prevalence of LVH has been reported, not related to blood pressure levels [42] and reversible to some degree after successful treatment [43]. It has been proposed that possible mediators for the effect of cortisol excess to LVH are hypertension, enhancement of noradrenalin and angiotensin II responsiveness and cardiomyocyte proliferation [44,45]. It must be noted though that concentrations of morning cortisol in all children were within normal limits and did not correlate to indices of adiposity, like previously reported [46]. Perhaps adverse effects occur at lower circulating levels of cortisol in obese children, which has to be clarified by further studies.

The type of pattern of LVH in obesity remains a field of controversy. The Bogalusa Heart Study in young adults showed that adulthood and childhood BMI were significant determinants of eccentric LV hypertrophy, while the presence of diabetes mellitus in adulthood and diastolic blood pressure in childhood predicted the development of concentric LVH [47]. In obese subjects with concurrent hypertension, concentric hypertrophy and concentric remodeling seem to be more prevalent [16,48]. Results in obese children and adolescents are inconclusive with some of them showing higher percentages of eccentric [10,49], while others of concentric type [50,51]. The prominent type of hypertrophy in our sample was eccentric which, in the context of absence of hypertension, can be interpreted as an adaptation to increased preload due to greater metabolic requirements, circulating blood volume and cardiac output, caused by excess adiposity [24].

Significant differences in diastolic function between groups, expressed by lower E/A ratio and higher E/e’, emerged in accordance with previous studies [21,22,52]. The ratio of peak early and late transmitral flow velocities E/A, correlated significantly with WHtR ratio and hs-CRP, but not with left ventricular mass like previously shown [53]. Both WHtR and hs-CRP are connected to visceral adiposity and low grade inflammation [54,55]. Waist-to-height ratio has been tested as a surrogate for abdominal obesity in adults and children and seems to be a better predictor of cardiometabolic risk than BMI [56,57]. High sensitivity CRP is a biomarker that quantifies low grade systemic inflammation, in the absence of overt systemic inflammatory or immunologic disorders and has been used as a predictor of cardiovascular events [58], but has been also associated with diastolic dysfunction and heart failure in adults [59,60,61]. In the study of Dahiya et al. in obese adolescents, CRP was an independent determinant of LV diastolic function [21]. Our study yielded significant association between hs-CRP in even younger children with shorter history of obesity. Thus it would be reasonable to suggest that increase in left ventricular filling pressures in obese children without further comorbidities are probably mediated by proinflammatory factors released by adipose tissue, rather than structural changes.

According to latest guidelines, TDI measurements are mandatory for the assessment of diastolic function in adults, since 3 of the 5 criteria are based on this method. Mitral annular diastolic velocity e’ is less load dependent than conventional transmitral flow velocities and by using E/e’ the effect of LV relaxation impairment on mitral E velocity is corrected [62]. Several pediatric studies incorporated TDI for the evaluation of diastolic function in obese children with conflicting results. Lambobarda et al. found no significant difference in E/e’ ratio between obese children and lean controls [17] and the same applies to the study of Barbosa et al. in which only higher velocities a’ of mitral annulus in late diastole were recorded [63]. On the contrary, Ghandi et al. reported a statistically significant dampening in E/e’ in the case group [52]. In the present study, E/e’ ratio was significantly higher in the obese group in accordance to previous studies [22,53,64] and correlated significantly with WHR, skinfold and uric acid levels when both groups were considered together. It must be noted that despite the significant differences between groups none of the children met adult criteria for diastolic dysfunction [62]. Nevertheless, these differences indicate subtle preclinical changes, which may persist and deteriorate into adulthood.

By using 3.16 as a cut off for HOMA-IR, about 40% of obese children showed impaired insulin sensitivity, presumably attributed to obesity as prepubertal children were selected in order to rule out as much as possible the confounding effect of puberty in insulin resistance. Insulin homeostasis model showed significant correlation to anthropometric indices and metabolic parameters. However, relation of HOMA IR to structural and functional cardiac parameters was not detected in our sample like previously reported [16,50,65].

There were certain limitations in our study. Our sample was relatively small but still statistically significant differences between the two groups emerged. Blood pressure levels were measured at a single visit which cannot rule out the masked hypertension and the white coat hypertension phenomena [66]. Assessment of LV by m-mode method is based on geometrical assumptions, but is considered fairly accurate in normally shaped ventricles. Thus m-mode is still recommended in the evaluation of LV mass and categorization into different geometric patterns [35]. It has been shown that reduced sensitivity to insulin can be present even before first signs of puberty and it is more pronounced in children with increased cardiometabolic risk [67], so selecting of prepubertal obese children cannot completely rule out the effect of insulin resistance. The cross sectional nature of the study does not allow conclusions about cause–effect relationships, however it seems that subclinical diastolic dysfunction is partly mediated by factors related to adipose tissue so further studies are needed to shed more light in the underlying pathophysiology and investigate the reversibility of these findings after weight loss.

## 5. Conclusions

Our study demonstrated that changes in geometry of left ventricle and diastolic function can be present in obese children even in the absence of hypertension. Moreover, parameters such as WHtR, hs-CRP and cortisol levels were predictors of these changes and could serve as possible indicators of obese children at greater cardiometabolic risk, requiring further echocardiographic evaluation. Future studies in larger samples could define proper cut off values of these easy to use parameters.

## Figures and Tables

**Table 1:** Demographic and anthropometric characteristics between non-overweight and obese children.

Parameters	NW (*n* = 26)	OB (*n* = 36)	*p*
Sex (Male/Female)	16/10	24/12	0.79
Age (years)	9.65 ± 1.57	9.76 ± 1.28	0.772
BMI (kg/m^2^)	17.09 ± 1.54	26.5 ± 3.43	0.000
BMI z score	0.11 ± 0.66	2.14 ± 0.32	0.000
NC (cm)	28.99 ± 1.93	33.29 ± 1.96	0.000
WHR	0.87 ± 0.05	0.93 ± 0.04	0.000
WHtR	0.44 ± 0.03	0.59 ± 0.05	0.000
Triceps fold (mm)	8.12 ± 2.43	20.28 ± 3.9	0.000
Subscapular fold (mm)	6.17 ± 1.62	20.46 ± 5.51	0.000
Suprailiac fold (mm)	5.95 ± 1.87	19.3 ± 4.04	0.000

Data are presented as means ± standards deviation. BMI—Body Mass Index; NC—Neck circumference; WHR—Waist to hip ratio; WHtR—Waist to height ratio.

**Table 2:** Comparison of biochemical data between non-overweight and obese children.

Parameters	Non Overweight (*n* = 26)	Overweight (*n* = 36)	*p*
UA (mg/dL)	3.29 ± 0.63	4.9 ± 1.04	0.000
TC (mg/dL)	163.59 ± 27.16	170.67 ± 38.26	0.452
HDL (mg/dL)	73.72 ± 20.59	54.53 ± 14.73	0.001
LDL (mg/dL)	77.18 ± 18.18	98.14 ± 28.93	0.004
TG (mg/dL)	64.59 ± 26.65	89.5 ± 45.50	0.023
hs-CRP(mg/dL)	0.04 ± 0.05	0.28 ± 0.19	0.000
HbA1c (%)	5.15 ± 0.31	5.26 ± 0.29	0.225
Cortisol (μg/dL)	9.44 ± 5.05	8.73 ± 4.93	0.601
HOMA-IR	1.66 ± 0.68	2.95 ± 1.51	0.000
Insulin (μIU/mL)	7.83 ± 3.27	14.4 ± 7.38	0.000

Data are presented as means ± standards deviation. UA—Uric Acid; TC—Total Cholesterol; HDL—High Density Lipoproteins; LDL—Low Density Lipoproteins, TG—Triglycerides; hs-CRP—high sensitivity C Reactive Protein; HbA1c-glycosylated hemoglobin; HOMA-IR—Homeostatic Model Assessment for Insulin Resistance.

**Table 3:** Comparison of echocardiographic measurements between non-overweight and obese children.

Parameters	NW (*n* = 26)	OB (*n* = 36)	*p*
SBP (mmHg)	101.15 ± 10.23	107.58 ± 7.84	0.07
DBP (mmHg)	64.35 ± 11.15	66.25 ± 7.7	0.43
LAD (mm)	24.7 ± 3.29	29.13 ± 3.44	0.000
IVSd (mm)	6.63 ± 0.67	8.05 ± 1.03	0.000
LVIDd (mm)	38.19 ± 4.05	42.64 ± 3.50	0.000
LVPWd (mm)	6.77 ± 0.68	8.26 ± 1.02	0.000
EF%	72.46 ± 5.05	72.85 ± 4.22	0.747
E(m/s)	1.00 ± 0,16	1.08 ± 0.20	0.124
A(m/s)	0.49 ± 0.09	0.62 ± 0.12	0.000
E/A	2.08 ± 0.56	1.76 ± 0.33	0.013
e’(m/s)	0.18 ± 0.03	0.18 ± 0.02	0.345
a’(m/s)	0.08 ± 0.16	0.08 ± 0.02	0.139
E/e’	5.43 ± 0.96	6.04 ± 1.13	0.032
LVM (gr)	70.19 ± 19.32	109.04 ± 31.94	0.000
LVMI	28.31 ± 6.22	40.05 ± 9.44	0.000

Data are presented as means ± standards deviation (SD). SBP—systolic blood pressure; DBP—diastolic blood pressure; LAD—left atrium diameter; IVSd—intraventricular septum thickness at end-diastole; LVIDd—left ventricular internal dimension at end-diastole; LVPWd—left ventricular posterior wall thickness at end-diastole; EF—ejection fraction; E/A—ratio of early to late diastolic transmitral flow peak velocity; e’—peak velocity of early diastolic mitral annular motion; a’—peak velocity of late diastolic mitral annular motion; E/e’—Peak velocity of early diastolic transmitral flow to peak velocity of early diastolic mitral annular motion; LVM—Left Ventricular Mass, LVMI—Left Ventricular Mass Index.

**Table 4:** Correlation between anthropometric and cardiometabolic factors and homeostatic model assessment of insulin resistance (HOMA-IR), left ventricular mass index (LVMI), E/A, E/e’.

Parameters	HOMA-IR		LVMI		E/A		E/e’	
	All	Obese	All	Obese	All	Obese	All	Obese
HOMA-IR	-	-	0.167	−0.028	−0.086	0.239	0.031	−0.074
LVMI	0.167	−0.028	-	-	−0.174	−0.083	0.175	−0.030
E/A	−0.086	0.239	−0.174	−0.083	-	-	0.236	0.277
E/e’	0.031	−0.074	0.175	−0.030	0.210	0.277	-	-
BMI	0.528 **	0.236	0.585 **	0.387 *	−0.369 **	−0.232	0.114	−0.146
NC	0.577 **	0.273	0.578 **	0.429 *	−0.287 *	−0.200	0.234	−0.104
WC	0.555 **	0.250	0.580 **	0.280	−0.376 **	−0.357 *	0.158	−0.126
WHR	0.187	−0.100	0.483 **	0.280	−0.275 *	−0.307	0.358 **	0.021
WHtR	0.412 **	−0.030	0.632 **	0.414 *	−0.439 **	−0.347 *	0.166	−0.091
Triceps	0.543 **	0.306	0.573 **	0.375 *	−0.377 **	−0.231	0.205	−0.047
Subscapular	0.525 **	0.299	0.534 **	0.205	−0.398 **	−0.272	0.288 **	−0.026
Suprailiac	0.545 **	0.335	0.590 **	0.333	−0.242	0.162	0.364 **	−0.053
SAP	0.319 *	0.477**	0.269 *	0.114	0.072	0.056	0.161	0.073
UA	0.628 **	0.263	0.447 **	0.021	−0.184	−0.028	0.346 **	0.050
TC	−0.131	−0.351	−0.100	−0.100	−0.079	−0.212	0.000	−0.048
HDL	−0.569 **	−0.252	−0.308 *	0.044	0.355 *	0.274	−0.096	0.329
LDL	0.199	−0.378	0.191	−0.050	−0.248	0.160	0.123	−0.030
TG	0.406 **	0.437*	0.039	−0.023	−0.188	−0.009	−0.015	−0.186
hs-CRP	0.708 **	0.002	0.510 **	0.232	0.510 **	0.232	0.122	−0.129
Cortisol	−0.088	−0.038	0.160	0.252	0.160	0.252	0.122	−0.084

** Correlation is significant at the 0.01 level; * Correlation is significant at the 0.05 level. BMI—Body Mass Index; NC—neck circumference; WHR—waist to hip ratio; WHtR—waist to height ratio; E/A—ratio of early to late diastolic transmitral flow peak velocity; LVMI—Left Ventricular Mass Index; E/e’—peak velocity of early diastolic transmitral flow to peak velocity of early diastolic mitral annular motion; SAP—systolic arterial pressure; HOMA-IR—homeostatic model assessment for insulin resistance; UA—uric acid; TC—total cholesterol; HDL—high density lipoproteins; LDL—low density lipoproteins, TG—triglycerides; hs-CRP—high sensitivity C reactive protein.

**Table 5:** Logistic regression analysis to predict LVH (*n* = 36).

Variables	Beta (OR)	S.E	*p*
Sex	−1.609 (0.2)	1.044	0.123
SBP	−0.053 (0.948)	0.057	0.354
Cortisol *	2.851 (17.305)	1.0209	0.018
WHtR	0.259 (1.296)	0.109	0.018

* values of cortisol were log transformed.
